# Factors associated with the implementation of health-promoting telework from the perspective of company decision makers after the first COVID-19 lockdown

**DOI:** 10.1007/s10389-022-01717-z

**Published:** 2022-05-04

**Authors:** Gert Lang, Kathrin Hofer-Fischanger

**Affiliations:** 1 Austrian Health Promotion Fund, Austrian National Public Health Institute, Aspernbrückengasse 2, 1020 Vienna, Austria; 2 FH JOANNEUM – University of Applied Sciences, Kaiser-Franz-Josef-Straße 24, 8344 Bad Gleichenberg, Austria

**Keywords:** COVID-19, Teleworking/working from home, Workplace health promotion, Technology acceptance model, Structural equation model

## Abstract

**Aim:**

Owing to the COVID-19 pandemic, many companies shifted to telework, with few insights into its implementation, organisational conditions or the role of workplace health promotion and management. This study focused on a multifactorial investigation of conditions in companies which implemented and evaluated telework during the first lockdown in 2020 as well as on their future intentions to facilitate teleworking under health-promoting working conditions.

**Subject and methods:**

The research hypotheses relate to an extended technology acceptance model. In a mixed-methods design, expert interviews were fed into the development of an online questionnaire. Out of 1858 contacted companies representing a broad range of Austrian businesses, 192 responses (general management, workplace health managers, etc.) were analysed using descriptive and multivariate statistics.

**Results:**

The degree of implementation and claim to teleworking increased significantly during the first lockdown and did not return to pre-pandemic levels afterwards. Changes depended on preparation and experience: evaluation of teleworking and willingness to continue offering teleworking were conditional on preparation and the degree of implementation. Prerequisites for future intentions to implement health-promoting teleworking included readiness, general willingness and existing workplace health promotion/management structures.

**Conclusion:**

This paper demonstrates the potential of health-promoting organisational cultures for development processes – particularly in times of crisis. Anchoring health-focused structures in companies helps to create health-promoting frameworks. Health-promoting teleworking can be developed from workplace health promotion/management using established approaches. It is essential to build on in-house capacities and competencies to develop awareness for a holistic culture for health-promoting (tele)work and to encourage deliberations about potential measures.

## Introduction

### Telework during the pandemic

Owing to the COVID-19 pandemic, many companies had to find rapid solutions in early 2020 so that their employees could continue to work – from home. In order to help contain the spread of the virus, allow employees to continue working and limit the economic consequences of the pandemic, many companies and employers shifted to teleworking (TW) if at all possible. What made such a radical transformation possible in the first place was existing digital information and communication technologies (ICT) in today’s increasingly flexible world (of work) (Emilie 2021; Poethke et al. 2019).

This was ‘(…) *an unprecedented, large-scale experiment in mass teleworking, and it seems likely that this expanded use of telework will not end with the end of the pandemic. This extraordinary situation brings both unprecedented opportunities and major challenges*’ (ILO 2021: 3). Telework, in the sense of working from an offsite location, is a concept which first appeared in the 1950s; since the 1980s, it has been repeatedly called ‘*the next workplace revolution*’ (Kelly 1985, quoted in Baruch 2001: 114). It may have increased in frequency since then but has certainly fallen well short of expectations, despite growing interest on the part of employers, employees and (e.g. the telecommunications) industry. Thus, between 2009 and 2019, only approximately 5.4% on average of employees in the EU-27 regularly worked from home (Milasi et al. 2020).

On the outbreak of the COVID-19 pandemic, TW reached new heights indeed. The International Labor Organisation called the dramatic developments a ‘*Teleworking “Tsunami”*’ (ILO 2021): on average 24% of employees in Europe who had never worked from home before began TW at this time, compared with 56% of employees who had at least occasionally worked from home before the pandemic. A European survey revealed that 48% of the employees who participated in the survey had worked at home during the COVID-19 pandemic for at least some of the time, while 34% only worked from home (Eurofound 2020). The data also show that the proportion of those working exclusively from home varied greatly by EU member country, e.g. when comparing southern, central, eastern or northern Europe (Vargas Llave 2021). Added to this, ‘*the preparedness for telework at a large scale is higher in ICT- and knowledge-intensive sectors, and generally for high-skilled workers, although with big differences across EU countries*’ (Milasi et al. 2020). Alongside economic structures and business sectors, differences such as the size of a company, organisation of work (autonomous, flexible work schedules) and employees’ qualifications (digital competencies) were decisive.

### Working conditions and health when teleworking

To date, there is no standard definition for telework (TW), as illustrated by the range of different terms used, such as *working from anywhere, telecommuting, working from home, mobile work, off-site working, remote working*, etc. They all have one factor in common, however, namely that the office is not (no longer) the only place where people do their work, although it took electronic aids (such as ICT) to make this possible. Thus, ‘*[t]eleworking occurs when employees perform all or a substantial part of their work physically separated from the location of their employer, using IT for operation and communication*’ (Baruch 2001: 114).

Even though many companies shifted to TW, this certainly does not mean that this shift was successful or that the conditions when working from home were conducive to good health. In particular, studies have reported on the impact of working from home on individuals’ physical, mental and social health (Lopez-Leon et al. 2020; Oakman et al. 2020; Tavares 2017). Work-space design, dealing with different types of technical equipment (e.g. VDU work) or a lack of personal skills (time management and self-management) can have a negative impact on physical health. This can result in impaired concentration, sleep disorders, headaches, gastrointestinal illnesses, excess weight and cardiovascular problems as well as musculoskeletal issues, particularly neck and shoulder pain (Badura et al. 2016; Elisson 2012; Tavares 2017; Thorp et al. 2011; Xiao et al. 2021). TW can have a negative effect on mental health if, for example, the organisational framework is not set up to be conducive to good health, resulting in mental issues such as feeling overwhelmed, exhausted or even depressed (Eurofound 2020; Oakman et al. 2020). In addition blurred boundaries between individuals’ private and professional lives can lead to family conflicts (Allen et al. 2015; Gajendran and Harrison 2007). Employees may perceive the feeling of also having to be available outside working hours as an additional burden (Pangert et al. 2016). This can lead to more stress, low spirits, sickness-related absenteeism and private conflicts (Minow and Swart 2019). The risk of social isolation as well as feelings of loneliness and fear can arise when employees have little contact with their co-workers or superiors and, at the same time, cannot compensate for that with family and friends (Badura et al. 2016; Lopez-Leon et al. 2020; Mann and Holdsworth 2003; Oakman et al. 2020).

Alongside negative impacts, TW can also have positive effects on health, mostly in relation to mental health. Employees working from home are more satisfied overall and feel less work-related stress such as pressure to perform and overwork (Oakman et al. 2020). It is easier to find a balance between work and family life, and employees are less exhausted (Gajendran and Harrison 2007). Owing to flexible working hours and fewer interruptions or distractions from co-workers, employees are more productive when working from home (Montreuil and Lippel 2003). Increased autonomy also increases job satisfaction, reduces labour turnover and guards against family conflicts. Thanks to less time spent commuting and increased flexibility time-wise, it is possible to enjoy a socially more active private life. This results in fewer conflicts between partners or other members of the family and more time to spend with the children (Hill et al. 2003; Lott 2020). Employees working from home have a healthier lifestyle overall, characterised by better sleeping habits, more exercise and coping better with stress (Grzywacz et al. 2007).

All things considered, the evidence presented so far suggests that TW has substantial health benefits for employees and economic advantages for companies. Nevertheless, the negative impacts presented above can cause health issues for employees (incl. managers) in the long term.

### Telework and workplace health promotion

As part of a company’s health policies or Workplace Health Management (WHM), Workplace Health Promotion (WHP) represents a high-quality concept. As a corporate or organisational development strategy, its goal is to improve health in the workplace, enhance well-being in the workforce and prevent ill health (ENWHP 1997; O’Donnell 2017). Health promotion relates to processes of participation and empowerment, with individuals taking control of those things which determine their health and well-being; its holistic approach has proven successful (WHO 1986). In this approach, health is created within the context of everyday life. Establishing working conditions which promote health in a company setting (WHP) is a key field of action for health promotion.

The core principles formulated in the ‘*Luxembourg Declaration on Workplace Health Promotion in the European Union*’ (ENWHP 1997) are considered to be the established guidelines for quality in WHP. They cover (1) incorporating WHP in all important decisions and in all areas of a company (integration), (2) involving the entire workforce at all levels in its conception and implementation (participation), (3) implementing environment-directed and individual-directed measures to reduce risks and improve safety factors and health potentials (holistic approach) and (4) introducing all measures systematically (project management).

Various quality assurance systems covering the processes and measures of WHP have been established in German-speaking countries, such as the Swiss ‘Friendly Work Space’ label or the German ‘Seal of Corporate Health’. Together with the Austrian Health Promotion Fund (Fonds Gesundes Österreich, FGÖ), the Austrian Network for Workplace Health Promotion (Österreichisches Netzwerk Betriebliche Gesundheitsförderung, ÖNBGF) already launched a quality assurance system in 2004. Companies are awarded a WHP quality certificate based on a standardised assessment system with a catalogue of 15 quality criteria for WHP (Heigl 2014), including the embedding of WHP in corporate principles/culture, the implementation of WHP structures (for projects), the identification of people responsible for WHP, workplace health management (WHM), communication, environment-directed and individual-directed measures (e.g. technical equipment). Companies with particularly successful approaches are awarded a WHP prize. Alongside their practical relevance in business practice, the quality criteria have also undergone a process of validation (Lang et al. 2019).

Several more recent, systematic reviews have attested the efficacy of WHP, especially when comprehensive (multimodal and holistic) programmes are implemented in relation to individual employees and their work environment for the purposes of promoting health (Allen et al. 2017; Ananthapavan et al. 2018; Brinkley et al. 2017; Goldgruber and Ahrens 2010; Hendren and Logomarsino 2017; Tam and Yeung 2018). A meta-analysis by van de Ven et al. (2020) revealed that WHP measures can be more effective when targeting lower socioeconomic groups.

Thus, WHP can play a decisive role when preparing and supporting companies and their employees for new challenges in the world of work and when adopting appropriate measures. This is particularly true in the current phase of radical transformation in the world of work (digitisation, flexibility, blurred boundaries, etc.) when key parameters are changing for health and work (Poethke et al. 2019). Although conditions in the workplace form the basis for healthy employees and competitive companies, health-promoting TW has not been paid much attention so far (Hager 2018). In the interests of modern organisational development and corporate strategies (for innovative, flexible companies) and in view of the prevalence of TW, the question as to the role of holistic WHP/WHM is becoming increasingly important in the implementation or continued development of telework which promotes good health.

Against this background, this paper addresses the following questions:

- To what extent did companies in Austria which were already familiar with health-promoting measures implement TW in the course of the first lockdown in 2020 and what did they gain from the experience?
- What factors are associated with the willingness of these companies to continue implementing TW and to improve its contribution to health promotion?
- What role do in-house health policies play in the process, i.e. WHM and WHP and the company’s readiness for the implementation of and experiences with TW?

First of all the theoretical foundations were laid and verifiable research hypotheses were derived. The study design combined qualitative data from focus groups with experts and quantitative data collected in a questionnaire sent to companies. A hypothesis-driven approach was used to analyse the quantitative data from the questionnaire with the help of univariate and multivariate methods. This paper discusses the main organisational structures and processes for implementing and expanding TW within a company. These insights help reconstruct the relevant processes for in-house decision making and acceptance. Suitable measures can then be derived to support and establish TW on a broad basis and in the sense of a holistic notion of health promotion.

## Study design and research methods

### Theoretical and methodological foundations

In his analysis of various frameworks, Baruch (2001) proposes adopting a broad theoretical perspective and distinguishes conceptually between four factors of TW namely the individual (personality, situation), the job (nature of work, technology), the organisation (strategy, culture) and nation (society). The implementation of TW can then be accounted for systematically, distinguishing between antecedents (feasibility, prior experience) and preconditions (culture, strategy, infrastructure) as well as processes (work/family balance, IT intensity) and the positive and negative outcomes of TW (health). Only a combination of factors and processes allows a comprehensive enquiry into the topic of TW. In this study, which aims to investigate a company’s disposition towards (health-promoting) TW, this requires paying special attention to organisational and operational levels and processes.

When endeavouring to explain the manner of implementing TW, approaches from behavioural theory have proven particularly fruitful. The conceptual framework of the theory of reasoned behaviour (TRA) and the theory of planned behaviour (TPB) (Ajzen 1991; Ajzen 2012), for example, starts from the premise that the behaviour of individuals (e.g. employees, managers) is basically guided by their motivation and intention to act and this is principally shaped by various factors on personal, social, organisational and contextual levels. Behavioural intention is the result of (a) an affective process of evaluation (attitude) or one’s belief that one’s behaviour leads to specific results, coupled with the evaluation of these results (e.g. expected benefits), (b) a subjective belief in what others expect from and approve of in the individual (social norm), as well as (c) difficulties subjectively perceived by the individual (self-efficacy) when behaving in a particular way (behaviour control). These theoretical assumptions help when clarifying reasons for TW, whereby especially beliefs influence attitudes and attitudes create intentions, which then regulate behaviour.

An influential narrower definition of these theoretical assumptions appeared in the guise of the technology acceptance model (TAM) (Davis 1989; Davis et al. 1989). Numerous literature reviews and meta-analyses do not only illustrate the broad application and applicability of this model but also attest to its usefulness (Abbas 2018; King and He 2006; Qingxiong and Liping 2004; Schepers and Wetzels 2007; Turner et al. 2010; Wu et al. 2011; Yousafzai et al. 2007a; Yousafzai et al. 2007b). Pérez Pérez et al. (2004, 2007) fleshed it out for TW, exploring the driving forces and barriers affecting the introduction and implementation of TW while taking account of a series of technological, human resources and organisational factors as possible determinants.

In line with the TAM approach, the first assumption is that the behavioural intention to implement TW is mainly determined by positive impressions of or attitudes towards making use of TW. Key in-house actors (e.g. managers, employees) identify aspects of organisational behaviour which they consider to be beneficial for job performance or useful for work. That is why attitudes towards TW are determined by such considerations of usefulness. What TAM has in common with TRA/TPB is that attitudes towards behaviour are fundamentally influenced by relevant beliefs. In accordance with TAM, a (positive) attitude towards TW is not only determined by perceived usefulness (PU) but also by the perceived user friendliness/useability of TW (perceived ease of use, PEOU). TW is not only determined by attitudes but also by the perceived usefulness of the degree of implementation.

These approaches and models (TAM in particular) serve as the theoretical foundation, albeit taking the following comments into account:

1. Because we are interested in the concrete experiences of working from home during the COVID-19 pandemic, instead of the perceived ease of use (PEOU), the degree of realisation of TW will be considered in the course of the first lockdown in 2020.
2. Following TAM, it is assumed that (successful) implementation goes hand in hand with a (positive) assessment of the efficiency, controllability and manageability of TW. Within a company or in the eyes of company decision makers, the perception of usefulness can be taken as an evaluation of TW – in the sense of how well or how poorly TW worked.
3. The attitude towards TW (ATT), in the sense of a motivational aspect, is understood as the willingness of the company to (continue to) implement TW or working from home.
4. The behavioural intention (BI) does not relate to the intention to implement TW in general but rather specifically to the behavioural intention of the company to implement health-promoting working from home.

The consideration of two additional (external) aspects (factors) makes it necessary to extend TAM. One of these factors is the organisational aspect of readiness, i.e. whether companies were operationally prepared to implement TW (so-called *operational telework readiness, RN*). Because teleworking ‘(…) *can be operationalized by the combination of technical and communication advances*’ (Shah and Manna 2020: 50), it is primarily associated here with requirements relating to technology and communication (so-called ICT). The other factor is about quality-assured company health policies (WHP & WHM) which, in the sense of a holistic understanding, take account of processes and measures to boost the well-being of a company and to promote the health of its employees. On a structural level, it is geared to establishing a health-promoting corporate culture as well as improving (organisational) working conditions, (technical) work equipment/resources, (social) communication and management (e.g. healthy management, remote digital management).

### Research hypotheses

**Fig. 1:**
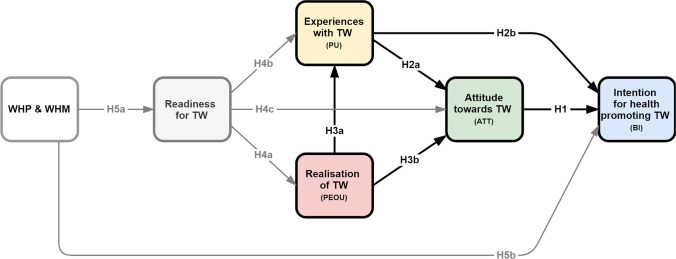
Conceptual (hypothetical) baseline and extended model. Notes: Directed arrows depict hypothesised effects between latent variables/factors; abbreviations in brackets symbolise variables from the Technology Acceptance Model (TAM): PEOU = perceived ease of use, PU = perceived usefulness, ATT = attitude towards behaviour, BI = behavioural intention; hypotheses H1 to H3 represent the baseline model; the extended model integrates H4 and H5 as well

The basic theoretical premise is that the stronger the behavioural intention to implement health-promoting TW (working from home), the more likely it is to happen.^1^ The more beneficial the direct and indirect spheres of influence are, the stronger, in turn, this intention is. The following research hypotheses (H1-5) and models (cf. Figure 1) were formulated in relation to the period under consideration in the COVID-19 pandemic, i.e. the first lockdown in 2020:

- (H1) The more positive the attitude towards TW (ATT), the stronger the intention to implement (health-promoting) TW in the company (BI).
- (H2) The better the company’s practical experiences with TW (PU), (a) the more positive the attitude towards TW (ATT) and (b) the stronger the company’s intention to set up health-promoting structures for TW (BI).
- (H3) The more intensively TW was implemented in the period under observation (PEOU), (a) the better the evaluation of its realisation (PU) and (b) the more positive the attitude towards TW (ATT) in the company.
- (H4) The better the company’s readiness for TW (RN), (a) the more comprehensive its implementation (PEOU) in the period under observation, (b) the better its evaluation (PU) and (c) the better the attitude towards TW (ATT).
- (H5) The stronger the anchoring of health-promoting corporate policy in the company (WHP & WHM), (a) the better the company’s readiness for TW (RN) and (b) the stronger the intention to set up (better) health-promoting structures for working from home in the future (BI).

### Survey instrument and operationalisation

An online workshop with 11 academics and practitioners (WHP/WHM consultants, the Austrian Network for WHP and researchers investigating the work environment) served as a qualitative step in the development of a standardised questionnaire. In a pre-test involving 10 people, the clarity of the survey instrument was checked as well as the order of items and how long it took to respond to them. In the introduction and questions section, health-promoting TW was defined as follows: ‘*Health-promoting telework is understood as taking account of different conditions which facilitate health-oriented working from home,* e.g. *complying with the company’s guidelines, creating an ergonomic and pleasant workspace, making the necessary hardware and software available, taking account of personnel resources and aspects of the sociospatial environment.*’

The operationalisations of the baseline model (incl. the reliability of the scales in the available sample) are as follows^2^:

- In connection with the amount of TW (PEOU), (a) the degree of TW allowed in the company expressed as the number of days per month per employee [degree] and (b) the group of people entitled to TW as a percentage of the workforce [claim] were taken account of in the period before, during and after lockdown (degree: α = 0.68; claim: α = 0.82).
- The perceived usefulness (PU) of TW was ascertained as experience with or an evaluation of the quality of the implementation of TW in the following item: ‘*How good or poor were the experiences that the company had with teleworking?*’ [experience] (with a bipolar response format: 1 = very poor, 5 = very good).
- The individual-directed attitude towards TW (ATT) was covered in the following item: ‘*How high or low is the general willingness in the company to make teleworking possible?*’ [willingness] (1 = very low, 5 = very high).
- Three variables were used to measure the intention to implement health-promoting working from home on a strategic and operative level (BI): ‘*How strong or weak is the company’s pursuit of the strategy of implementing or expanding health-promoting telework?*’ (1 = very weak, 5 = very strong) [int1], ‘*How likely or unlikely is* (…)’ [int2] and ‘*How large or small is the company’s intention to implement the guidelines in the ‘Handbook for health-promoting telework’?*’ (1 = very unlikely/small, 5 = very likely/large) [int3] (α = 0.85).

In the extended model, the following external variables were included:

- In order to assess the company’s readiness (RN) for TW, two variables were considered: ‘*How good or poor was the company’s technical readiness for teleworking?*’ [rn1] and ‘(…) *was the company’s adaptation of communication channels in relation to teleworking?*’ [rn2] (both 1 = very poor, 5 = very good) (α = 0.82).
- The structural and normative embedding of a health-promoting corporate culture was operationalised in three variables: ‘*How strong or weak is the embedding of WHP or WHM in the company?*’ [whp1] (1 = very weak, 5 = very strong), ‘*How useful or useless do you* (…)’ [whp2] or ‘(…) *other people in the company assess WHP or WHM to be for the company?*’ [whp3] (1 = very useless, 5 = very useful) (α = 0.63).

Finally, details were gathered on the company (size, branch and sector, award: WHP quality certificate/prize) and on the person filling out the questionnaire for the company (sex, age, education, position in the company).

### Data basis

The selected population was composed of decision makers in Austrian companies who had run a funded WHP project in their companies or who had taken part in a WHP training course and whose e-mail addresses had been saved in a contact database. In the course of the first lockdown in early 2020, a link to an existing ‘*Handbook for health-promoting telework*’ (Kappel and Hofer-Fischanger 2019) was sent to *N* = 1858 company representatives by e-mail (using the MailJet program) to provide support to companies during the pandemic and in September they were asked to participate in a standardised online questionnaire (LimeSurvey, V3.16.1). A total of 146 (7.9%) e-mails could not be delivered. Of the adjusted sample of *N* = 1712 (1858-146), 270 companies which had implemented TW responded but 78 (4.6%) were excluded due to unit non-response (<50.0% of the questionnaire filled in). In total *n* = 192 were included in the analysis, which represent 11.2% of the adjusted selected population. Details of the sample are given in Table 1.

**Table 1:** Characteristics of the companies and respondents

Companies	Categories	n	%	Respondents	Categories	n	%
Company size (*n* = 184)	Small business (10-49 employees)	44	20.9	Sex (n = 187)	Female	136	72.7
	Medium-sized business (50-249 employees)	54	29.3		Male	51	26.6
	Large business (>250 employees)	86	46.7	Age (n = 187)	Up to 29 years	15	8.0
Federal province (*n* = 181)	Eastern Austria (Burgenland, Lower Austria, Vienna)	78	43.0		30-39 years	44	23.5
	Southern Austria (Carinthia, Styria)	44	24.3		40-49 years	45	24.1
	Western Austria (Upper Austria, Salzburg, Tirol, Vorarlberg)	59	32.6		50-59 years	72	38.5
Branch (*n* = 183)	Health & social services	47	25.7		60 years or older	11	5.9
	Public administration, defence, social insurance institutions	33	18.0	Education (n = 185)	Apprenticeship, vocational school	14	7.5
	Various services^1^	29	15.8		School-leaving examination (academic secondary school, vocational college)	42	22.6
	Education	19	10.4		University of applied sciences, university	129	69.4
	Manufacturing	14	7.7	Position/area of responsibility (*n* = 303)^4^	WHP/WHM	110	57.3
	Wholesale and retail trade	10	5.5		Personnel (HRM)	48	25.5
	Other^2^	31	16.9		Management	36	19.1
Sector (*n* = 174)	Private	56	32.2		Occupational safety & health^5^	25	13.3
	Public	70	40.2		Employee representative	18	9.6
	Non-profit	39	22.4		Quality management	15	8.0
	Other	9	5.2		Other	51	27.1
WHP award^3^ (n = 192)	No/not specified	74	38.5	Working hours (n = 187)	Up to 20 h/week	16	12.3
	WHP quality certificate	103	53.6		Up to 38 h/week	81	43.3
	WHP prize	15	7.8		More than 38 h/week	90	44.4

^1^ Finance/insurance, professional/scientific/technical, other (commercial) services, ^2^ Information/communication, arts/entertainment/recreation, energy supply, construction, transportation/storage, mining, water supply, international organisations/bodies, accommodation/food and beverages, ^3^ variables combined from several items, ^4^ multiple response set: the given percentages relate to the number of valid cases, ^5^ incl. Representatives for safety, disabled persons and equal opportunities

A dropout analysis was possible for various characteristics, with the percentage differences between the obtained sample and the population being negligible for the sex of respondents (women: +2.9%, men: −2.4%, do not know: −0.5%) and somewhat larger for the geographical location of the company (by federal province, ranging from −5.5% for Styria to +5.8% for Lower Austria) as well as for its economic branch/sector (using NACE^3^ ranging from −4.2% for health and social services to +7.3% for public administration, defense and social insurance institutions).

### Data analysis

Several steps were carried out in the statistical analysis. The data gathered in the questionnaire was analysed descriptively (SPSS 26) using the mean (m), standard deviation (sd), skew (s^3^) and kurtosis (s^4^). The expectation was that the variables would demonstrate significant dispersion around the mean (i.e. differentiation) and that the items would not be skewed (s^3^ < |2.0|) and would have unimodal distribution (s^4^ = |7.0|) (Byrne 2012), i.e. without floor or ceiling effects, making them suitable for multivariate data analysis.

The hypotheses were examined with the help of structural equation models (SEM) with latent variables in Mplus 7 (Muthén and Muthén 2012). When data follow an approximately normal distribution and sample sizes are small (*n* ≤ 200), the maximum likelihood estimation method estimates undistorted model parameters (Curran et al. 1996). First the baseline model and then the extended model was specified; for identification purposes one factor loading per latent variable was fixed at 1. The validity and reliability of the measurement models were investigated using the following criteria: standardised indicator validity (λ > 0.50) and reliability (R^2^ > 0.25) (Bagozzi and Baumgartner 1994) as well as internal consistency reliability (Cronbach’s alpha α ≥ 0.60) (Robinson et al. 1991). The hypotheses were tested using importance and statistical significance (*p* ≤ 0.05) of the standardised effect coefficients (γ or β). Absolute and comparative fit indices serve to evaluate the goodness of fit of the models: χ^2^ test (χ^2^/degrees of freedom (df) < 3.0), Root Mean Standard Error of Approximation (RMSEA<0.08), Comparative Fit Index/Tucker–Lewis Index (CFI/TLI > 0.90) and Standardised Root Mean Square Residual (sRMR<0.08) (Browne and Cudeck 1993; Hu and Bentler 1999; Kline 2011). The modification indices were considered where necessary to improve the model, e.g. plausible measurement error covariances.

## Results

### Implementation of and experiences with telework

**Fig. 2:**
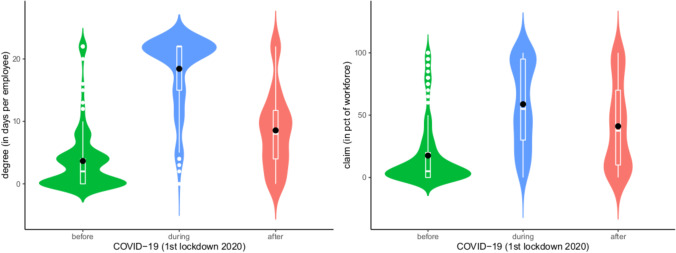
Degree of teleworking allowed in the company (*n* = 187) and claim to teleworking accepted by the company (*n* > 166). Notes: The figures show violin plots, including box-and-whisker plots and arithmetic means (points)

Both the degree to which TW was made possible in companies and the percentage of the workforce which could lay claim to TW increased significantly during the first lockdown and did not return to pre-pandemic levels after it ended (degree before: mean m = 3.64, during: m = 18.41 and after the first lockdown: m = 8.58 days per month/employee; claim before: mean m = 17.44%, during: m = 58.67%, after: m = 40.81% as a percentage of the workforce; cf. Fig. 2).^4^

As far as their experiences are concerned, the companies evaluated the implementation of TW during this period as being ‘fairly good’ on average, i.e. as having greater usefulness (m = 4.05). In addition, the companies reported that their preparations for TW, from both a technical and communicative perspective, were good on average (m = 3.27 and m = 3.23, respectively). The company’s willingness to continue with TW was assessed as being ‘average’ to ‘fairly high’ (m = 3.47). On a scale of 1 = very weak to 5 = very strong, the goal of strategically anchoring health-promoting TW in companies had a mean of m = 2.77. The probability of and intention to use the guidelines pertaining to health-promoting measures for TW was somewhat better on average (m = 3.07 and m = 2.93 respectively). At the time of the survey, anchoring WHP and WHM within the company was considered by the company representatives to be ‘fairly strong’ (m = 3.71). WHP/WHM were assessed by the respondents themselves as being ‘fairly useful’ to ‘very useful’ (m = 4.53) and by other people in the company as being ‘fairly useful’ (m = 3.62).

It needs to be emphasised that some of the variables scattered widely around the reported mean, displaying rather good distribution properties (e.g. low skew, no ceiling or floor effects). Detailed descriptive statistics and correlations between variables and factors are provided in Table 2.

**Table 2:** Descriptive statistics and correlations

Factors	Variable (no.)	Descriptive statistics (*n* > 146)					Correlations^a^ (*n* ≥ 135)											
		m	md	s	s^3^	s^4^	(1)	(2)	(3)	(4)	(5)	(6)	(7)	(8)	(9)	(10)	(11)	(12)
WHP & WHM	whp1 (1)	3.71	4.00	1.07	−.68	−.14	1											
	whp2 (2)	4.53	5.00	.73	−1.53	1.87	.31*	1										
	whp3 (3)	3.62	4.00	.88	−.06	−.71	.43*	.37*	1									
Readiness for TW	rn1 (4)	3.27	3.00	1.26	−.38	−.83	.26*	.03	.14	1								
	rn2 (5)	3.23	3.00	1.17	−.32	−.76	.29*	.06	.26*	.70*	1							
Realisation of TW	degree^b^ (6)	13.49	14.75	5.10	−.52	.03	.12	.03	−.04	.12	.21*	1						
	claim^b^ (7)	49.71	50.00	31.44	.17	−1.25	.03	.05	−.01	.13	.18*	.38*	1					
Usefulness of TW	experience (8)	4.05	4.00	.72	−.36	−.12	.05	.07	.09	.08	.24*	.06	.20*	1				
Attitude towards TW	willingness (9)	3.47	4.00	1.10	−.52	−.22	.19*	.05	.22*	.36*	.43*	.17*	.29*	.32*	1			
Intention to introduce h-p TW	int1 (10)	2.77	3.00	1.10	.12	−.64	.36*	−.06	.31*	.34*	.46*	.27*	.33*	.18*	.41*	1		
	int2 (11)	3.07	3.00	1.10	−.14	−.45	.15	.11	.24*	.21*	.31*	−.01	.08	.06	.28*	.48*	1	
	int3 (12)	2.93	3.00	1.08	−.16	−.43	.25*	.04	.22*	.29*	.37*	−.01	.08	.07	.39*	.60*	.84*	1

n, number of valid cases; m, mean; md, median; s, standard deviation; s^3^, skewness; s^4^, kurtosis; WHP & WHM, Workplace Health Promotion & Management; TW, teleworking; ^a^ Pearson’s correlation coefficients after pairwise deletion of missing cases (* *p* ≤ 0.05); ^b^ The index value includes the average degree of or average claim to telework during and after the first lockdown

### Factors associated with (health-promoting) teleworking

In the baseline model, a significant positive effect (γ = 0.25) was estimated for the realisation of TW on the evaluation of TW. In other words, the better the perceived experience with or evaluation of TW, the higher the degree of TW allowed and the greater the claim to TW during and after the first lockdown. In addition, the company’s willingness to continue to implement TW did not only increase in relation to a higher realisation of TW during and after the first lockdown (β = 0.30) but also with a positive experience with or evaluation of the usefulness of TW (β = 0.26 | both *p* ≤ 0.01) in the course of the first lockdown. The intention to implement health-promoting TW (working from home) was only associated with a general willingness (i.e. motivation) for TW in the company (β = 0.53 | *p* ≤ 0.001) but not by the evaluation of TW implemented during and after the first lockdown (b = 0.03 | *p* > 0.05).

**Fig. 3:**
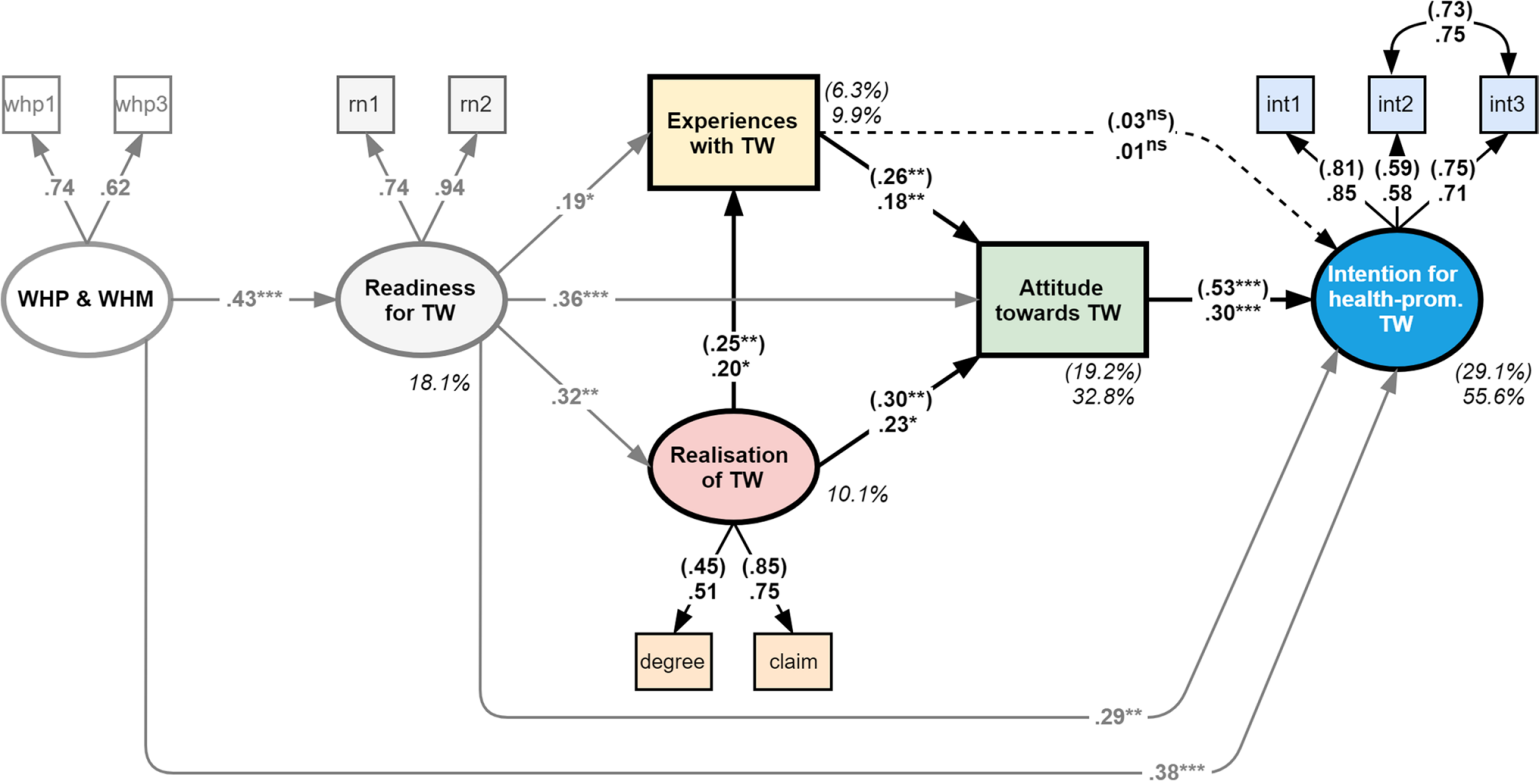
Results of the baseline and extended models. Notes: *n* = 185 (pairwise deletion of missing cases); single-headed arrows depict effects between variables, double-headed arrows depict error covariance; rectangles symbolise variables and ellipses factors with factor loadings significant at *p* ≤ 0.001; all coefficients are standardised (maximum likelihood estimation), incl. explained variances of dependent variables (%); numbers in parentheses show the results of the baseline model, numbers without parentheses those for the extended model; fit of the baseline model: χ^2^(df) = 22.719(10), χ^2^/df = 2.272, RMSEA = 0.083, P-close = 0.105, CFI = 0.963, TLI = 0.923, SRMR = 0.053; fit of the extended model: χ^2^(df) = 48.474(34), χ2/df = 1.426, RMSEA = 0.048, P-close = 0.521, CFI = 0.976, TLI = 0.961, SRMR = 0.044; significance levels: *p ≤ 0.05, **p ≤ 0.01, ***p ≤ 0.001, ^ns^ not significant

The goodness-of-fit of the empirical data in relation to the baseline model is seen to be good because all fit statistics fulfil the required threshold values. The baseline model can explain between 6.3% and 29.1% of the dependent variables (cf. Fig. 3).

Building on the specifications of the baseline model, the extended model takes additional external determinants (TW readiness and WHP & WHM) into account. The estimates reproduced the structural effects of the baseline model throughout, albeit partially to a somewhat lesser extent. The newly added latent variable of TW readiness confirmed the assumed relationships: companies which had prepared for TW operationally were not only in a position to realise it to a greater extent during and after the first lockdown (γ = 0.32) and had better experiences (higher perceived usefulness) with TW as implemented in this period (γ = 0.19) but also proved to have a higher general willingness to continue to implement TW in the company (γ = 0.36). According to the modification index, there was an additional specific effect of TW readiness on behavioural intention (β = 0.29): the better companies were prepared for TW, the stronger their intention to set up health-promoting conditions for TW in the future. This intention was also influenced decisively by the company’s health policies in two different ways: More strongly anchored or better experiences with WHP/WHM did not only mean that the company was considerably better prepared for TW (β = 0.43) but also that a stronger intention emerged to set up (better) health-promoting structures for working from home in the future (β = 0.38) (both *p* ≤ 0.05).

All factor loadings on the specified latent variables in the extended model were within an acceptable range, although the variable [whp2] was not included in the extended model due to its factor loading being too low (λ < 0.50). The model accuracy of the extended model is excellent and is able to reproduce the empirical observations very well. The relatively few variables in the model explain the relevant proportions of variance in the dependent variables, such as 55.6% of the intention to implement health-promoting TW/working from home (Fig. 3).

## Discussion

This paper investigates key factors contributing to the acceptance or initiation of the decision-making process regarding (health-promoting) TW in the course of the first COVID-19 lockdown. The willingness to implement TW taking account of health-promoting working conditions was inspired by the model of technology acceptance (TAM), an approach which explains the use of technology, and extended by two key aspects.

Taking Austrian companies which were already familiar with health-promoting measures as an example, it was possible to demonstrate that the degree of TW increased significantly during the first lockdown and did not return to pre-pandemic levels after it ended. The companies which participated in the survey were well prepared, both technically and in relation to communication channels, and evaluated TW as being useful overall. Many companies were willing to continue making use of TW to a medium or high degree. Other studies have revealed similar results on the degree and use of TW as well as willingness to retain it in the future (e.g. Alipour et al. 2020).

For the most part, the study was able to confirm the assumptions set up in the theoretical model in relation to a sample of Austrian companies which had allowed TW right from the beginning of the pandemic. Using the overall model it was generally possible to reconstruct processes relevant for in-house acceptance and decision making relating to (health-promoting) TW during the first lockdown. Especially the high explanatory power of the intention to implement health-promoting TW (55.6%) is a particularly impressive result in view of it being such a complex phenomenon.

It turned out that the realised extent of implementation of TW in a company was a key factor for both the evaluated quality of the experience with TW and the company’s willingness to continue implementing TW (after the first lockdown as well). Given the cross-sectional design of the study, it might well be that the perceived earlier quality of TW was also already a determinant of whether TW was implemented. However, this demonstrates that cognitive, affective, motivational and conative aspects are all meaningful for the implementation of TW or its continued development with a focus on promoting health. A key prerequisite for the implementation and decision-making processes turned out to be operational readiness for the degree of and claim to TW during and after the first lockdown, which replicates more recent findings in the field (Shah and Manna 2020). Readiness was a decisive precondition in order to ensure that work could still be carried out with the help of ICTs as well as in a radically different environment (i.e. TW) in the full lockdown phase. Operational readiness (existing or recently initiated) in relation to technical requirements and communication channels facilitated a higher degree of implementation during the period in question, contributing to a more positive report on experiences with TW (e.g. due to its smoother implementation). This was associated with the company’s willingness to offer (health-promoting) TW in the future. A company’s organisational structures and normative anchoring of WHP/WHM also proved to be a fundamental or favourable precondition for its intention to facilitate TW in the future with a special emphasis on health-promoting factors.

This confirms what Baruch (2001) already identified as being an essential organisational factor for feasible or effective TW: alongside the type of work and technical suitability of specific tasks, a supportive corporate culture and willingness is indispensable. While it was technology that first allowed work to be decoupled from the office, both social and organisational factors are associated with the willingness to make use of TW. In addition to providing the necessary hardware and software, training is essential to pass on basic knowledge and ICT skills, as are specific competencies such as self-sufficiency, confidence and communication (cf. Pérez Pérez et al. 2004, 2007).

One of the key prerequisites for successful TW relates to a company’s leadership and management, which has to create appropriate norms and corporate culture. Culture determines collective organisational and individual behaviour in organisations and affects all areas of a company for management and employees alike (decision-making processes, management, social relationships, communication, etc.). Organisational culture is described as ‘(…) *the collection of traditions, values, policies, beliefs and attitudes that constitute a pervasive context for everything we do and think in an organisation*’ (Marshall and McLean 1985). According to Schein (2010), as a pool of collective beliefs, values and rules, organisational culture does not only have a significant impact on the operational readiness and commitment of an organisation but also on the performance of its members, i.e. on their well-being and health as well (Badura et al. 2016).

In relation to WHP/WHM, an organisational culture and climate which promotes health defines organisational qualities, e.g. the extent to which health and well-being are laid down in corporate governance and management systems (key corporate documents, guidelines), are structurally anchored within the company, are the responsibility of specific groups, have pre-defined contact persons with specific areas of responsibility and, finally, are defined for the purpose of achieving them in appropriate forms of cooperation (empowerment, participation) and specific (environment-directed and individual-directed) measures.

The COVID-19 lockdown represented a changed environment which demanded a swift transition to TW so as to allow companies to continue to do their work. When they represent a structurally anchored and a normatively sustained health-promoting corporate culture, WHP/WHM turned out to be an enabler or motivator in processes of organisational transformation or development. Companies with stronger health structures and collectives (health culture) had stronger intentions to set up better health-promoting structures for working from home in the future. In this respect, being structurally, normatively and operationally equipped for change has impacts on thinking, feelings, motivation and behaviour with associated consequences that promote or hamper health and well-being.

## Methodological limitations

The results presented in this paper should be interpreted against a background of methodological limitations. Owing to its cross-sectional design and the nature of the data, correlations can be established but, technically speaking, it is not possible to draw direct conclusions in relation to the direction of causal effects and the potential for reverse causality should certainly not be ignored. Even though the factors under investigation could not be taken into consideration in an appropriate longitudinal design, it is important to stress that the causal chains postulated here are certainly plausible in theoretical terms. The requirements of causality (Pearl 2000) were addressed with retrospective and prospective formulations of questions (Moosbrugger and Kelava 2020) and variables were sorted based on insights from research into attitudes and behaviour (Aiken 2002).

In relation to the values and thresholds acceptable in the field, the models used were parsimonious and the identified factors had good validity and reliability. Nevertheless, measures with lower reliabilities (e.g. α = 0.63) are unsatisfactory. In addition operationalising factors with only one indicator should be avoided in future studies because it is not possible to evaluate the quality of measurement (Bollen 1989). Even though the actual manner of implementing health-promoting TW could not be ascertained but just the intention to do so, a meta-analysis of TAM approaches suggests that there is a positive correlation between intentions and actions (Turner et al. 2010). Although inspired by the TRA, the original TAM does not include any social norms. Venkatesh and Davis (2000) already took this into account, while the review by Legris et al. (2003) and the meta-analysis by Schepers and Wetzels (2007) support the normative reference in processes of change. This extension of the model also proved successful in our approach in relation to WHP/WHM culture.

In addition, company decision makers were asked to share their views, which could represent only a limited approximation of the organisational perspective. Moreover, the survey took place in September 2020. Because the items related to the period of the first lockdown from March to April 2020, this could have led to misleading information due to imperfect powers of recall.

Finally, several aspects regarding representivity need to be considered because the companies were not selected on the basis of random sampling but were deliberately chosen for their previous and varying experiences with WHP/WHM. Since WHP and WHM are not evenly distributed across sectors, the results of this study cannot be directly applied to all companies in Austria. The sample in this study covered a broad range of branches but due to the lower response rate of the study, selection bias is possible and cannot be entirely ruled out on the basis of the provided dropout analysis. Nevertheless, the different response rates for some branches suggest that TW was not possible to the same extent everywhere or was even impossible in some sectors. The results of another study very similar to ours showed that more than 90% of large companies in Austria implemented TW in 2020, and it was highest in sectors such as information and communication, finance and insurance, education and public administration (Bachmayer and Klotz 2021).

## Conclusions and implications for practice

To cope with economic and health-related challenges, the pandemic made it necessary to introduce changes in the way companies organised work, including a rapid and comprehensive switch to TW. Adaptations to organisational structures (e.g. ICT) and workflows were imperative and necessary capacities relating to that had to be built up (e.g. knowledge, competencies, qualifications). Companies which were prepared found themselves at an advantage during the first lockdown in 2020. Positive experiences with TW have been continuously consolidated since then and many companies will continue TW in the future. With the expanded and prolonged use of TW, company representatives and employees are increasingly recognizing the need for health-promoting conditions when working from home. In this respect, the findings of this study provide a useful blueprint for essential structures and processes that need to be adapted or set up in a company in order to be prepared for similar contextual changes in working conditions, such as future lockdowns.

The switch to or intensification of TW can be associated with health risks. It has proven useful to anchor a health-promoting culture within a company. This can cover crucial organisational determinants of health and well-being in the workplace (Zwetsloot and Leka 2010). WHP/WHM also supports health-promoting measures for TW and can influence the decisions and practices of individuals and organisations. It seems to be the case that ‘*[t]he more rapid the changes in society and the more turbulent the business climate for an organisation, the more important its internal cohesion thanks to commitments to common beliefs, values and rules*’ (Badura et al. 2016: 93).

WHP and WHM are well established concepts which foster a targeted, longer-term development of structures, processes and general conditions in companies going beyond a single project cycle. Established approaches can be drawn on and divided into individual steps, e.g. initiation, diagnosis, planning, implementation, monitoring, evaluation and sustainability. Only broad-based involvement of different stakeholders within a company such as management, personnel and organisational development, change management, IT, occupational safety and health (WHP/WHM) and employee representatives (works council) can bring about a long-term cultural change in the company. It is not enough for individual employees to accept change. Instead everybody needs to be involved constructively in the process of organisational development, including a shift in the right direction (Schepers and Wetzels 2007). That is important because when introducing and using (digitally supported) ICT (like TW, for example) the reciprocal links between technological, social, organisational and work-related dimensions should be taken into account in a complementary fashion rather than just concentrating on the technology (Hirsch-Kreinsen and Wienzek 2019). The scope for a particular set-up is not to be found in individual parts of the system (technology, organisation, work) but rather in the interdependencies or interfaces between different elements in the system.

Health promoters who intend to implement and monitor a shift in an organisation are also key figures when it comes to implementing health-promoting TW successfully. The first step involves gathering information on the conditions in the company relating to TW as well as on the culture of the organisation; here initial analyses can be very informative. The unique nature of the setting and characteristics of groups of employees have to be taken into account. For example, (e.g. ICT) interventions can differ for various groups of (e.g. younger vs. older) employees in relation to their needs (e.g. digital (health) competencies), requirements (e.g. communication), opportunities (e.g. usability) and willingness (e.g. when faced with processes of change). In order to avoid an economic and sociostructural digital divide, WHP must contribute to more equal opportunities health-wise and counteract the development of social inequality. Differences in competencies between large, medium-sized and small businesses or between different groups of employees should be accommodated in the process (Hirsch-Kreinsen and Wienzek 2019).

The organisational development of health-promoting TW also requires the expansion of in-house capacities and competencies. Suitable training opportunities are needed for managers and employees to develop an awareness for a holistic culture of health-promoting (tele)work and to boost considerations of measures in the companies where they work. For example, workshops on self-management and time management, virtual and hybrid cooperation or ‘remote management and leadership’ could be on the programme. Measures such as permanently staffed help desks, in-house training programmes for all employees or guidelines for implementing holistic health-promoting TW prompt sustainable developments in the sense of holistic WHP/WHM.

## Data Availability

Not applicable.

## Footnotes

1 For general TAM, a systematic review (Turner et al. 2010) was able to confirm the basic premise that behavioural intention correlates with actual use.

2 Due to the cross-sectional design, it was not possible to take account of whether health-promoting TW was actually implemented after the questionnaire was submitted (e.g. by the end of 2020).

3 NACE is the classification of economic activities in the European Union (cf. https://ec.europa.eu/eurostat/statistics-explained/index.php?title=NACE_background).

4 Because we are interested in the impacts of the COVID-19 pandemic, a separate index was created for further use for the degree of and claim to TW and the means were calculated for during and after the lockdown.
